# Epstein-Barr virus associated hepatic smooth muscle tumor in a patient with acquired immunodeficiency syndrome: A case report

**DOI:** 10.1097/MD.0000000000019930

**Published:** 2020-05-01

**Authors:** Qihui Zhou, Fengtian Wu, Yongzheng Guo, Biao Zhu

**Affiliations:** Department of Infectious Diseases, State Key Laboratory for Diagnosis and Treatment of Infectious Diseases, National Clinical Research Center for Infectious Diseases, Collaborative Innovation Center for Diagnosis and Treatment of Infectious Diseases, the First Affiliated Hospital, College of Medicine, Zhejiang University, Hangzhou, Zhejiang Province, China.

**Keywords:** acquired immune deficiency syndrome, case report, epstein-barr virus, liver, smooth muscle tumor

## Abstract

**Introduction:**

Epstein-Barr virus (EBV) associated smooth muscle tumors (SMTs) usually present under the condition of immunosuppression, including congenital immunodeficiency syndrome-SMT, post-transplantation-SMT and HIV-SMT. HIV-SMTs are most likely to invade the central nervous system, followed by the liver, lungs, and other locations.^[1]^ Many laboratory techniques, including serological techniques, polymerase chain reaction and immunohistochemistry (IHC), are employed to determine the aetiologies of these tumours. With respect to therapy, surgical resection is the main treatment. In patients with immunodeficiency, improving immune status is significant for defending against other viruses. We describe a case of the primary focus of SMT in the liver of HIV-positive patient without any metastasis.

**Patient concerns:**

A young male HIV-positive patient complained of fever and abdominal pain for 2 months.

**Diagnosis:**

IHC of liver tissue confirmed the finding: EBV-related smooth muscle tumor.

**Interventions:**

Given the patient's general condition, he was not a suitable candidate for surgical resection. He was given antibiotics, antifungal agents and EBV-directed agents to control infection as well as highly active antiretroviral therapy to enhance the immunity.

**Outcomes:**

The patient's symptoms improved. He was discharged.

**Conclusions:**

In conclusion, EBV-related HIV-SMTs is a rare neoplasm found in the liver among immunodeficient patients. This case highlights that a variety of examinations such as IHC for smooth muscle markers (smooth muscle actin and desmin) and EBER, as well as polymerase chain reaction for EBV DNA should be done when diagnoses are ambiguous.

## 1 Introduction

Epstein-Barr virus (EBV) associated smooth muscle tumors (SMTs) usually present under the condition of immunosuppression, including congenital immunodeficiency syndrome-SMT, post-transplantation-SMT and HIV-SMT. HIV-SMTs are most likely to invade the central nervous system (CNS), followed by the liver, lungs, and other locations.^[1]^ Many laboratory techniques, including serological techniques, polymerase chain reaction (PCR) and immunohistochemistry (IHC), are employed to determine the aetiologies of these tumours. IHC confirmed positive staining for alpha-smooth muscle actin (SMA), and nuclear staining for EBV-encoded RNA-1 (EBER-1).^[2]^ Such tumours must be differentiated from various lesions with the same appearance, including Kaposi's sarcoma, myopericytoma, and gastrointestinal stromal cell tumour (GIST).^[3]^ With respect to therapy, surgical resection is the main treatment. In patients with immunodeficiency, improving immune status is significant for defending against other viruses. More beneficial and targeted treatments will be employed in the near future. Here, we describe a case of the primary focus of SMT in the liver of HIV-positive patient without any metastasis.

## 2 Case presentation

A twenty-seven-year-old man complained of fever and abdominal pain for more than two months. He went to a local hospital for treatment previously. Abdominal computed tomography (CT) revealed multiple low-density shadows in the liver; abscesses were first considered. Blood culture showed that *Cryptococcus neoformans* was present. Therefore, the patient was treated for several weeks with amphotericin B, fluconazole, and flucytosine to combat *C neoformans* and with antibiotics, including imipenem and cilastatin, to combat infection. He was diagnosed with HIV infection. His absolute CD4 T lymphocyte count was 39 cells/μl, and he received highly active antiretroviral therapy (HAART) consisting of tenofovir, lamivudine and efavirenz. Because his body temperature and abdominal pain were not well controlled, he was admitted to our hospital for further treatment.

On examination, the patient was pale and thin, with a body mass index of 16.9 kg/m^2^. His temperature was 38.2°C, heart rate was 106bpm, respiratory rate was 18 breaths per minute, blood pressure was 140/84mmHg and oxygen saturation in room air was 96%. His abdomen was scaphoid. His enlarged liver was palpable. Given prior abdominal CT findings for the patient, we empirically treated him with 500 mg of meropenem every eight hours and 400 mg of moxifloxacin each day. Considering the patient's symptoms and blood culture results, we concluded that he had cryptococcal septicaemia and administered 400 mg fluconazole each day for anti-fungal treatment. Given the patient's low CD4 counts, one daily tablet of sulfamethoxazole was added to his treatment regimen to prevent opportunistic infections. He also received HAART consisting of tenofovir, lamivudine and nevirapine.

The patient repeatedly exhibited high fever. Due to this manifestation as well as blood culture results, a spinal puncture was performed the day after the patient was admitted to our hospital. His intracranial pressure was 240 mm H_2_O. Routine and biochemical examinations of the cerebrospinal fluid produced findings within normal ranges. The count of nucleated cells was 0/μL. Two *C.neoformans* specimens were found on a prepared Chinese ink-dyed glass slide. Cerebrospinal fluid culture revealed no bacteria. A routine blood test showed moderate anaemia with a haemoglobin level of 7 g/dL and a leucocyte count of 3900/μL. Blood biochemistry tests showed an albumin level of 32.1 g/L and abnormal liver function, including an alkaline phosphatase level of 250 U/L and a glutamyltranspeptidase level of 555 U/L. The patient's erythrocyte sedimentation rate was 78 mm/h, and his high-sensitivity C-reactive protein level was 81.8 mg/L. Viral loads were undetectable for both plasma human cytomegalovirus and EBV. EBV serology was negative for anti-EBV viral capsid antigen IgM and positive for anti-EBV viral capsid antigen IgG.

**Figure 1:**
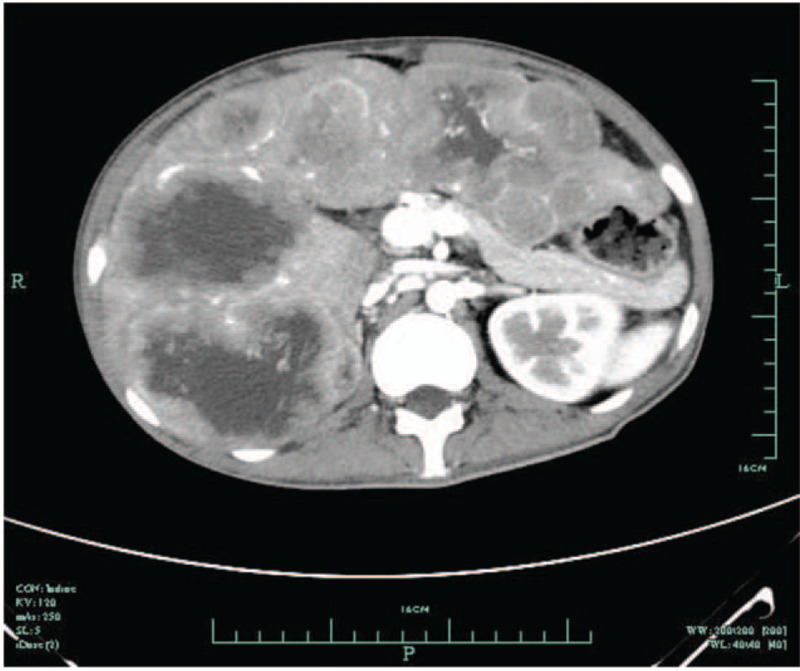
Abdominal computed tomography image. There were multiple sizes of intrahepatic abscesses, and enlarged lymph nodes around the mesenterium and posterior peritoneum as well as ascites.

**Figure 2:**
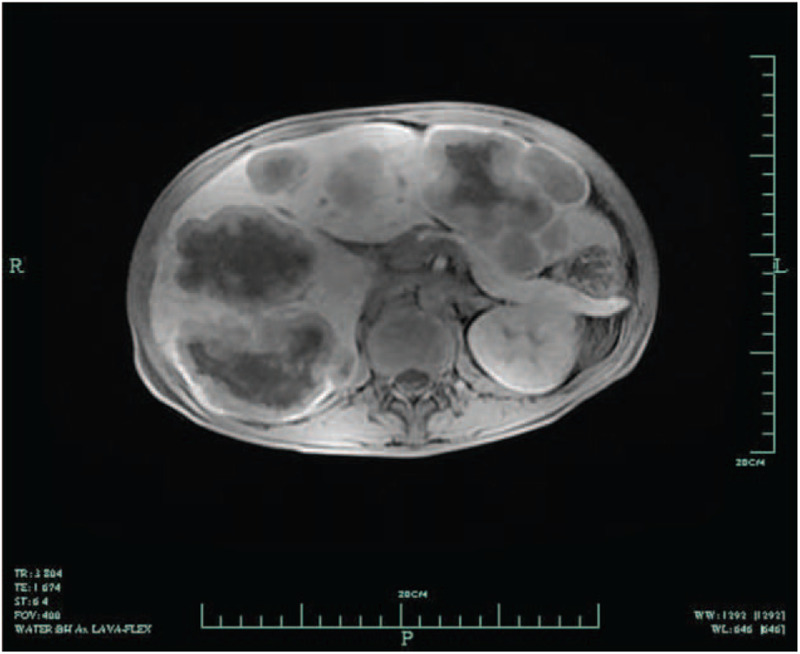
Liver magnetic resonance imaging. There were multiple intrahepatic lesions; abscesses and metastatic tumours were first considered. More enlarged lymph nodes were seen in the peritoneal and mesenterium areas.

**Figure 3:**
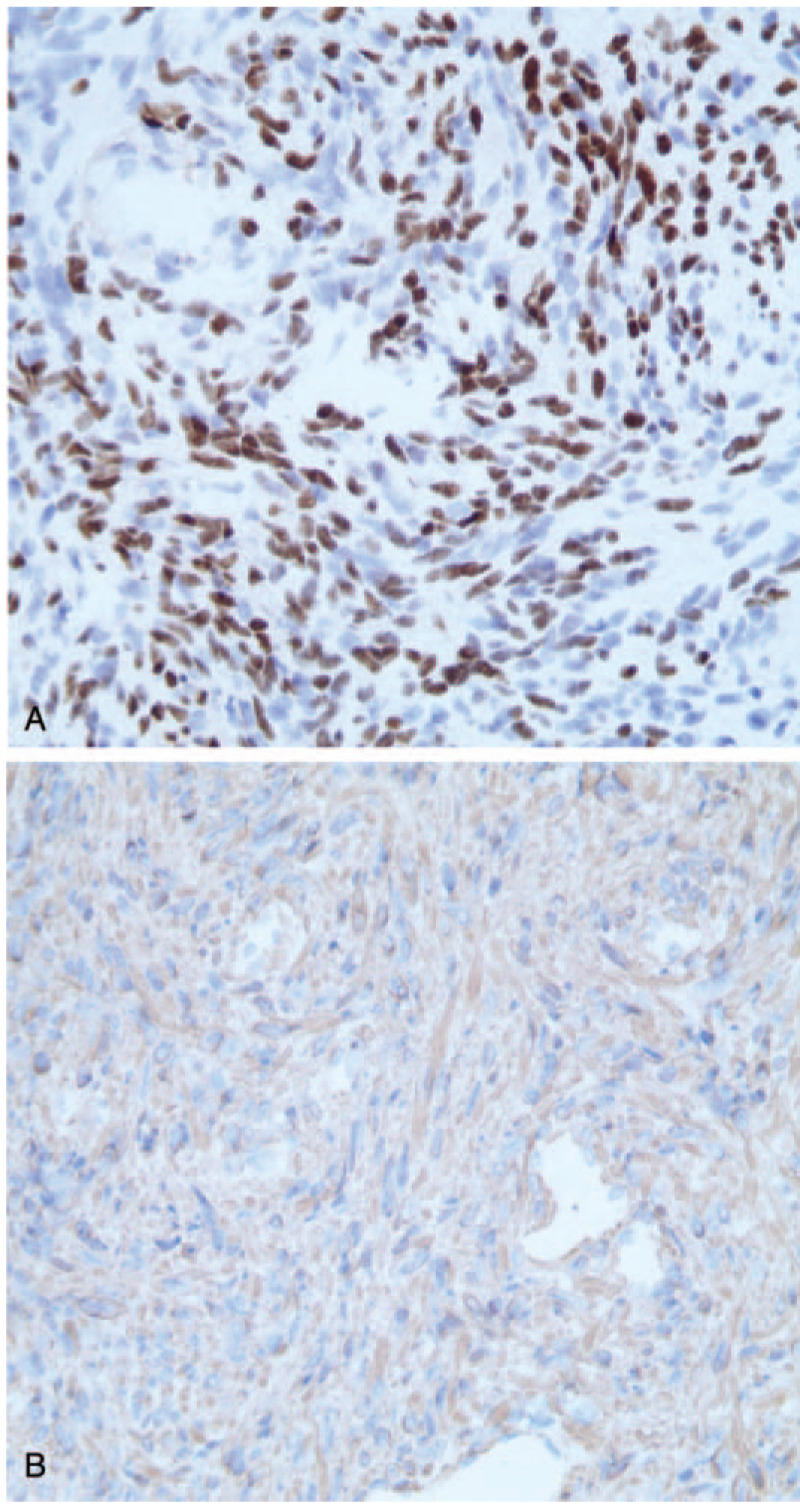
Immunohistochemistry. A: Immunohistochemical stains positive for Epstein-Barr virus-encoded RNA-1 (×400); B: Immunohistochemical stains positive for alpha-smooth muscle actin (×400). RNA = ribose nucleic acid.

On the fourth day after admission, abdominal CT showed multiple intrahepatic abscesses, and there were enlarged lymph nodes around the mesenterium and posterior peritoneum as well as ascites (Fig. 1). Liver magnetic resonance imaging showed multiple intrahepatic lesions; abscesses and metastatic tumours were first considered (Fig. 2). Comparisons of findings from these imaging examinations and from the abdominal CT performed at the prior hospital revealed no decrease in lesion sizes; therefore, liver puncture and catheterization were performed. The drainage fluid had a pellucid appearance, which we did not anticipate. Culture results indicated that neither fungi nor bacteria were present. Tumour cells could not be found. A routine drainage liquid test revealed 0 nucleated cells/μL. These tests were conducted several times and produced the same results. We then decided to perform a liver biopsy. Ultimately, pathological results unveiled the true cause of the observed findings: a soft tissue tumour of spindle cells. IHC confirmed positive staining for alpha-SMA (Fig. 3) and negative staining for CD117, CD21, CD34, DOG-1, S-100, Desmin, and human herpesvirus-8 (HHV-8). The tumours showed nuclear staining for EBER-1, which indicated that they were EBV-associated SMT (Fig. 3).

Given the patient's general condition, he was not a suitable candidate for surgical resection. The patient continued receiving HAART regimens to inhibit virus production and repair his immune system as well as EBV-directed agents. He was discharged. During follow-up, he expressed his preference to avoid surgical resection.

The patient was admitted to local hospital for further symptomatic treatments. He was badly ill and characterized by cachexia while in the hospital. His temperature fluctuated recurrently, and the highest temperature was 39.6°C. Abdominal pain could not be eased, but aggravated day by day. At the end of May 2017, the patient manifested as a few black rashes towering above skin at the corner of mouth and eyelids. His C-reactive protein level was 149.2 mg/L. The procalcitonin level was 8.9 ug/L. His absolute CD4 T lymphocyte count was 16 cells/μL. As the local hospital was unable to perform certain tests, there was no exact etiological evidence. He was given combined medication including anti-nontuberculosis mycobacteria, anti-talaromyces marneffei, anti-fungal and anti-viral therapies empirically. After a few weeks of treatments, his temperature finally returned to the normal range. At early morning on January 6th 2018, the patient was unconscious. He was given a series of emergency rescue measures at once. Family members did not want him to suffer continuously, they gave up further treatments. This HIV-positive patient died of multiple severe infections and advanced tumor.

## 3 Discussion and conclusions

EBV-related SMTs, including HIV-SMT, CI-SMT, and PT-SMT, have been reported in immunosuppressed patients. EBV-SMTs are prone to occur in female patients over 30 years old.^[4]^ SMTs can be classified into two categories, leiomyomas and leiomyosarcomas, which represent 3.8% and less than 10% of soft tissue tumours, respectively. There have been efforts to distinguish between benign and malignant tumours by utilizing histological features such as cytological atypia, cellularity, and necrosis. Compared with post-transplantation -SMT patients, HIV-SMT patients seem to have a higher mitotic rate.^[5]^ Mitotic activity is usually regarded as the most valuable predictive factor.^[4,6]^ Previous reports showed that leiomyomas behave like leiomyosarcomas in survival times and tumor spread. There is no definite association between histologic grade and prognosis.^[5,7]^ Certain reports collectively call them SMT due to indolentness.^[8]^ Their evolution is less rapid than we had expected. Sometimes, properties of SMTs vary, and are related to patients’ immune status, as HIV-positive patients have a worse prognosis than patients after transplantation.^[5]^

EBV, which is a member of the gamma subfamily of herpesviruses, plays a pivotal aetiological role in the occurrence of soft-muscle tumours in HIV-positive patients, especially the ones with CD4 counts below 100/mm^3^.^[9,10]^ This role is consistent with the roles of viruses in other associations between opportunistic oncogenic infections and cancers, including correlations between HHV-8 and Kaposi's sarcoma; EBV and non-Hodgkin lymphomas as well as nasopharyngeal carcinoma; human papillomavirus (HPV) and anogenital cancers; and hepatitis B/C and liver cancers.^[3,11]^ In contrast, EBV is rarely associated with SMTs in immunocompetent patients. HIV-SMT multiplicity is common, with an inclination to the CNS, including cranial and spinal epidural locations, and can arise in extra-CNS locations such as the liver, lung, vocal cords and adrenal glands.^[2,12]^ Metastasis is rarely seen. Many precise methods used to detect EBV genetic material include determinations of EBV seropositivity, PCR for EBV DNA, IHC for latent membrane protein-1 and EBV-encoded small RNA (EBER), as well as histopathology.^[2]^ Histologically, it is composed of two types of cells from well-differentiated monomorphic spindle cells to incomplete differentiated primitive ovoid cells.^[8,13]^ The features are similar to myopericytoma or angioleiomyoma.^[10]^ Typical diagnosis depends on IHC for smooth muscle markers (SMA and desmin) and EBER as well as PCR for EBV DNA. There are several explanations of the association between EBV and tumourigenicity of SMT. One explanation suggests that EBV attaches to smooth muscle cells via EBV receptor CD21 since normal smooth muscle cells weakly express EBV receptor CD21, as observed in this case, whereas SMT cells with EBV strongly express CD21. The combination of EBV and its receptor further induces CD21 expression, and this cycle repeats. Certain authors have suggested cell fusion between EBV-infected lymphocytes and smooth muscle cells.^[3,14]^ Fusion of EBV-positive lymphocytes with nonlymphoid cells that do not express CD21 has been demonstrated in vitro.

The molecular mechanism of SMT tumourigenesis is reactivation of the Akt/mammalian target of rapamycin (mTOR) signalling pathway. Studies by Sodhi^[15]^ et al discovered the aforementioned association. Similarly, Shen^[16]^ et al observed the nuclear staining of p-AKT and p-mTOR in most tumour cells, suggesting the activation of the Akt/mTOR signalling pathway.^[16]^ Since mTOR plays such a key role in mediating cell growth, sirolimus, an inhibitor of mTOR, could repress the progression of SMT, and EBV-related SMT and Kaposi sarcoma have been successfully treated using this approach. However, in our study, no nuclear staining for p-Akt (serine 473)/p-mTOR (serine 2448) was performed.

With respect to the differential diagnosis of spindle cell neoplasms in HIV-positive patients, IHC should be used to exclude Kaposi sarcoma, myopericytoma, GIST, and mycobacterial spindle cell pseudotumour. Negativity for endothelial cell markers CD31, CD34 and HHV-8 eliminates Kaposi sarcoma. The fact that antibody react to pathogen of Kaposi's sarcoma HHV-8 is not seen in EBV-SMTs.^[4]^ The absence of a haemangiopericytomatous growth pattern and the presence of desmin exclude myopericytoma. The absence of CD117 (ckit) and CD34, along with atypical clinical manifestations, eliminates GIST.^[3]^ A lack of acid-fast bacilli in tumour cells and the absence of CD68 may exclude mycobacterial spindle-cell pseudotumour.^[4]^

Regarding therapies for HIV-SMTs, Yin^[17]^ et al reviewed literature addressing therapeutic strategies and concluded that since there are currently no clinical or randomized controlled trials, it may be best to treat each patient in a case-by-case manner until a larger study is performed. EBV-SMTs are multifocal but are not a systemic disease or metastases, and the tumours are resistant to cytotoxic chemotherapy; surgical resection is the main treatment approach.^[2,14]^ As previously discussed, antiviral drugs such as acyclovir or ganciclovir used to suppress EBV infection and improve T-cell immunity are significant, and antiretroviral regimens HAART should be employed as a premise.^[14]^ Other potential options include Akt/mTOR inhibitors such as sirolimus and EBV-targeted T-cell therapies.^[4]^

In conclusion, EBV-related HIV-SMTs is a rare neoplasm found in the liver among immunodeficient patients. Diagnosis depends on IHC for smooth muscle markers (SMA and desmin) and EBER as well as PCR for EBV DNA. We hope that more beneficial and targeted treatments will be developed in the near future.

## Acknowledgment

Authors are grateful to the Department of Infectious Diseases, State Key Laboratory for Diagnosis and Treatment of Infectious Diseases, National Clinical Research Center for Infectious Diseases, Collaborative Innovation Center for Diagnosis and Treatment of Infectious Diseases, the First Affiliated Hospital, College of Medicine, Zhejiang University for their contribution in diagnostic evaluation. We are also grateful to Hui Ye, Feifei Su and all physicians from Department of Infectious Diseases of Wenzhou Centre Hospital for their contribution in providing the follow-up information of this patient.

## Author contributions

Qihui Zhou wrote the manuscript; Fengtian Wu and Yongzheng Guo collected the data; Biao Zhu proofread the mauscript.

Biao Zhu orcid: 0000-0001-6288-4575.
